# The Poor Man's Home.—XVI

**Published:** 1897-12-04

**Authors:** 


### Modern Sociology.

### THE POOR MAN'S HOME.—XVI.

### Housing in France—(*concluded*).

The better paid class of artisans, and also a good many clerks, find good houses provided for them by the "Working Men's Dwellings Company of Passy-Auteuil." Passy and Auteuil are two suburbs of Paris, contiguous to each other, and connected with the city by rail, boat, omnibus, and tramway. It is therefore convenient, while the air is purer than in the cheaper parts of the metropolis. The company began as a semi-philanthropic association, and dividends were to be limited to 4 per cent, As a matter of fact the utmost that has yet been paid has been 2½ per cent. per annum. The original intention was to sell the houses built by the company on the instalment plan, and tenants were not accepted who did not intend to purchase. This has now been so far modified that houses are rented to persons who do not intend to buy, in the hope that they will finally incline to do so; if they show no such intention they are told that the houses are required for others, presumably persons more amenable to this "Sam Slick" method of persuasion. The houses are one-storey cottages, built of different coloured bricks, and attractive in appearance. There are 64 in all, of various sizes. We shall speak only of the smaller ones. These contain four rooms; a dining-room of good size, 14 ft. 9 in. long by 9 ft. 5½ in. wide, behind which is a kitchen measuring 6 ft. 3½ in. in length, and the same width as the dining-room. One of the marvels of France, to one of another nation, is the microscopic dimensions of the average kitchen, and the good cooking that can come out of it. The two bedrooms are of the same size, and are nearly square—10½ ft. by 10 ft. 2½ in. A 3 ft. wide passage runs from front to back, so there is through ventilation. The water-closet and laundry are at the back of the house, and completely outside it. All the plumber work has been carried out on good sanitary principles. A cooking range is provided in the kitchen, and fireplaces in the other rooms. If the tenant desires it, the company will put gas-pipes through the house, and supply a gas cooking-stove and one bracket. The rent of such a house as this is 300 francs (£12), while in the neighbourhood half as much again is asked for similar accommodation. If the tenant intends to buy a house, he must put down a sum of 500 francs (£20) in the first place, and after that an amount equal to 8 per cent. of the cost for 20 years. Of this one-half is reckoned as rent, while the remainder is considered an instalment of the purchase money. Purchasers are not allowed to sell or let the property within the first ten years of their occupancy without the permission of the company. Subletting and taking lodgers also is forbidden, and no one is permitted to use a house as a shop or a public-house. If a tenant wishes to sell he must give the company the option of purchasing.

The Banque d'Escompte has also built in Paris some houses of the same character, but while great forbearance is shown in the case of tenants in arrear, the venture is more remunerative, and from 4½ to 6 per cent. has been paid. One cannot but wonder if the difference in result comes from the fact that the managers of the bank's property are better men of business than the others.

In the town of Havre there is a company of this kind, which owns 117 houses. This company was fortunate in buying its ground cheap, and it is now worth nearly four times as much as was paid for it. The Havre houses, like the Paris ones, are of four rooms, but they have a large kitchen meant to be also a living room, and three bedrooms. The kitchen and one bedroom measure 16 ft. 9 in. by 10 ft. 10 in., the other two rooms are 9½ ft. by 8½ ft. The rent charge is 430 francs (£17 4s.) a year, but this includes a part of the purchase-money. Ten per cent. on the cost is charged, and the house becomes the tenant's own property at the end of fourteen years. The sanitary arrangements of these houses are of a very primitive character, but, such as they are, they seem to be popular. The majority of the houses are sold, and the company can pay a dividend of 4 per cent., and have all the sums paid towards purchase for reinvestment. The rents are cheap, as rents go among the poor; that is, they take only about 15 per cent. of the earnings of the heads of families. A tenant who has purchased his house is not allowed to sell it within ten years; neither may he add to its height, nor build out on the space intended for a garden. He is forbidden also to use his dwelling as a public-house. Thus, although sold, the houses still remain under the control and subject to the rules of the company. In the case of a tenant failing twice consecutively to pay the instalments of the purchase-money as they fall due, the house may be sold over his head.

In France, a very large number of commercial firms have provided houses for their workpeople, the custom having begun with some of them as early as the middle of the eighteenth century. In these enterprises the employer, as a rule, seeks no gain beyond obtaining a good class of workers, who will appreciate the comforts they possess. The Familistère is the best known of these; but from the fact that it strikes at the root of family privacy, which is, perhaps, more valued in France than even in England, we do not consider it one of the best. M. Menier, of chocolate renown, is the owner of 295 comfortable and well-arranged tenements, the rent of which is 150 francs (£6) per annum. This represents about a twelfth of the earnings of the head of the house; but as women, and children over fourteen years of age, are also employed at the factory, the rent forms an unusually small item in the budget of M. Menier's employés.

